# Association between invasive cancer of the cervix and HIV-1 infection in Tanzania: the need for dual screening

**DOI:** 10.1186/1471-2458-8-262

**Published:** 2008-07-30

**Authors:** Crispin Kahesa, Julius Mwaiselage, Henry R Wabinga, Twalib Ngoma, Joan N Kalyango, Charles AS Karamagi

**Affiliations:** 1 Ocean Road Cancer Research Institute, P.O.Box 3592, Dar es Salaam, Tanzania; 2 Department of Pathology, Makerere University, P.O.Box 7072, Kampala, Uganda; 3 Clinical Epidemiology Unit, Makerere University, P.O.Box 7072, Kampala, Uganda; 4 Department of Pharmacy, Makerere University, P.O.Box 7072, Kampala, Uganda; 5 Department of Paediatrics and Child Health, Makerere University, P.O.Box 7072, Kampala, Uganda

## Abstract

**Background:**

Cancer of the cervix is the second commonest malignancy in females worldwide and is the leading malignancy among women in Tanzania. Cancer of the cervix has been strongly associated with Human Papilloma Virus (HPV) which is a sexually transmitted disease. However, the role of HIV-1 in the aetiology of cancer of the cervix is less clear. Studies suggest that HPV and HIV-1 infection are synergistic and therefore their dual occurrence may fuel increased incidence of cancer of the cervix and AIDS. We therefore conducted a study to determine the association between cancer of the cervix and HIV-1.

**Methods:**

The study was carried out in Ocean Road Cancer Institute, Dar-es-salaam, Tanzania between January and March 2007. A hospital-based case control design was used to study 138 cases and 138 controls. The cases were consenting women 18 years and above with histologically confirmed squamous cell carcinoma of the cervix, while the controls were consenting non-cancer adult women attendants or visitors. The participants were counselled and tested for HIV-1 and interviewed to assess risk factors for cancer of the cervix and HIV-1. Estimation of risk was done by computing odds ratios and confidence intervals. Confounding and interaction between the factors were assessed using logistic regression.

**Results:**

HIV-1 prevalence was much higher among the cases (21.0%) than among the controls (11.6%). In logistic regression, HIV-1 was associated with cancer of the cervix (OR = 2.9, 95% CI = 1.4–5.9). Among the cases the mean age was lower for HIV-1 infected (44.3 years) than HIV-1 uninfected women (54 years, p = 0.0001).

**Conclusion:**

HIV-1 infection is associated with invasive cancer of the cervix. Resource-constrained countries with a high burden of HIV-1 and cervical cancer should adopt a high-risk approach that targets HIV-1 positive women for screening of cervical cancer initially by utilizing HIV/AIDS resources.

## Background

Cervical cancer is the most frequent cancer of women in sub-Saharan Africa and the most common cause of cancer-related mortality [1]. The age-standardized incidence of cervical cancer in sub-Saharan Africa is 30 to 67 per 100,000, which is two to ten times higher than that in developed countries [1]. Unlike in developed countries where the incidence of cervical cancer has declined, the incidence of cervical cancer in sub-Saharan Africa has not decreased but has even risen in some regions [1,2].

The majority of cervical cancer cases can be prevented by screening and countries that have high coverage of cervical cancer screening have reduced invasive cervical cancer incidence by about 70–90% [3]. Although about 40–90% of women in developed countries are screened for cervical cancer [4-6], less than5% of women in developing countries undergo cervical cancer screening [7,8]. In a situational analysis for cervical cancer diagnosis and treatment in the East, Central and Southern African countries, Chirenje and colleagues found that, though 95% of health care facilities had the infrastructure for cervical screening, very few women were screened due to lack of policy guidelines, infrequent supply of basic materials, and lack of suitably qualified staff [7].

Cervical cancer is associated with many risk factors including early sexual debut, having multiple sexual partners or having sex with someone who has multiple sexual partners, being HIV positive, a family history of cervical cancer, older women, smoking and poverty [7,9-11]. By far the most important risk factor for cervical cancer is genital infection with human papillomavirus (HPV) which is responsible for virtually all cases [12,13]. There are many types of HPV classified as low-risk types (LR) that are usually found in non-malignant lesions, and high-risk (HR) types that are associated with cervical cancer [14,15]. HPV 16 and 18 are the most frequent types and cause about 70% of all cervical cancers worldwide. However, studies from sub-Saharan Africa suggest that other HR-HPV types are more prevalent and more diverse than elsewhere [16-19]. The reasons for the unusual pattern of HPV in sub-Saharan Africa are unclear but may be linked to HIV [20,21].

Since 1993, the US Centres for Disease Control and Prevention has included cervical cancer as one of the AIDS-defining illnesses [22]. Several studies conducted in developed countries have demonstrated an increased risk of invasive cervical cancer among HIV positive women [23,24]. In sub-Saharan Africa, the situation is less clear with conflicting results on the association between HIV and invasive cervical cancer [17,25-34]. In part, this confusion may explain the current situation in many countries in sub-Saharan Africa whereby the prevention and control of cervical cancer is not linked to that of HIV. Tanzania, a low-resourced country in East Africa, with a heavy burden of cervical cancer and a prevalence of HIV of 8%, is no exception. The aim of the study was therefore to link the twin burdens of cervical cancer and HIV by establishing an association between invasive cancer of the cervix and HIV-1 in Tanzania.

## Methods

The study was carried out at Ocean Road Cancer Institute (ORCI) in Dar-es-salaam, Tanzania between January and March 2007. ORCI was established in 1996 and is the only specialized centre for cancer treatment handling both referral and self referral cancer patients from all over Tanzania. It offers screening and treatment for cervical cancer and HIV as well as palliative care. ORCI has four wards with a total of 122 beds; two wards are for female patients, one for male and another ward for children. About 2500 new cancer cases are seen every year, two thirds of whom are women. Cervical cancer cases comprise 60–65% of new female cancer cases. ORCI was chosen because it is the only centre in Tanzania which provides specialized treatment for cancer including chemotherapy, radiotherapy and also has cancer specialists.

The study was a hospital-based case-control design. The cases were women aged 18 years or more, with newly diagnosed histologically confirmed cancer of the cervix, who attended ORCI between January and March 2007 and consented to participate in the study as well as to HIV testing. They were recruited from the out and in-patient departments and enrolled consecutively. The controls were women aged 18 years or more, without cancer, who attended ORCI between January and March 2007, and consented to participate in the study as well as to HIV testing. The controls were selected randomly from women who had accompanied or come to visit patients or women attendants of sick children within one week of selecting the cases. We selected these women rather than other patients in ORCI as controls because we wanted the controls to be as close as possible to "community controls". Patients who were too sick or unable to communicate in the language of the interviewer were excluded from the study.

Cervical cancer was confirmed on the basis of histological results of cervical specimens. Three cervical specimens from all new suspected cervical cancer patients were taken and put in a 5 ml bottle containing formalin. The specimens were then sent to Histology Department for examination. The pathologist issued a report confirming the histological diagnosis of cervical cancer. Among the controls, 80% agreed to undergo screening for cervical cancer and were found to be negative for invasive cancer of the cervix. No details of pre-invasive disease among the controls were available.

HIV serology was performed on cases and controls with two consecutive capillus tests confirmed with commercial ELISA (anti- HIV-1 micro Behringwerke, Marbug, Germany) after HIV counselling. The principal investigator with the help of two trained research assistants used a pre-tested structured questionnaire to collect data on social demographic characteristics (age, marital status, parity, education level, occupation, region of residence, zone of residence), reproductive factors (parity of woman, age at first pregnancy, age at first coitus, use of oral contraceptives) and smoking. The questionnaire was translated into Swahili in a standard procedure of translation and back-translation.

Data was entered into EPIINFO version 6.04 and then exported to SPSS version 13 for analysis. Continuous variables were summarized into means and standard deviation. Categorical variables were summarized into frequencies and percentages. The Chi-square test was used to compare categorical variables while the student's t test was used for continuous variables. Association between HIV and cancer of the cervix was established using Chi square tests and Fisher's exact test. Odds ratios were used to measure the strength of the association. All factors that were statistically significant at a p value of 0.2 or less at bivariate analysis were considered for multivariate analysis.

Multivariate logistic regression was used to assess for interaction and confounding. Product terms were formed between the main predictor variable (HIV-1 infection, categorized into HIV+ and HIV-) and other variables (age, parity, education, occupation, cigarette smoking and religion). We used the chunk test to see if there was interaction by comparing the -2LL (negative two log likelihood) of the reduced model (no interaction terms) and the full model (with interaction terms). We found no significant difference between these two models and concluded that there was no interaction. All variables were tested for confounding since they were not effect modifiers. We noted the odds ratio for HIV-1 when each of the variables was left out of the model and also when it was put back in the model. The variables that changed the odds ratio of HIV-1 by more than 10% were considered confounders. The variables were tested singly and also as groups of confounders. All statistical tests were two-sided.

Permission to carry out the study was obtained from Makerere Clinical Epidemiology Unit, Faculty of Medicine Research and Ethics Committee, Makerere University School of Graduate Studies, Ocean Road Cancer Institute Ethics Committee, and the National Institute of Medical Research (NIMR) Tanzania. Written informed consent to participate in the study and consent for HIV testing were obtained from the participants. Participants who were found to be HIV-1 positive were assisted to attend HIV clinics as per national guidelines.

## Results

A total of 145 cases and 152 controls were enrolled in the study. Seven cases and 14 controls were excluded because of refusal to test for HIV or language barrier. Thus 138 cases and 138 controls were available for final analysis. Overall, 18 (6.5%) of the participants were from southern zone, 27 (9.8%) were from central zone, 30 (10.9%) were from southern highland, 42 (15.2%) from lake zone, 111 (40.2%) from eastern zone and 48 (17.4%) were from northern zone. The mean age (SD) of the study participants was 50.0 (12.3) years and was significantly higher among the cases compared to the controls (51.9 yrs vs. 47.4 yrs; p-value = 0.002). The proportion of cases compared to controls decreased with increasing education level (Chi square for trend = 12.654, p < 0.001). There was no significant difference between cases and controls with respect to occupation, residence, marital status and religion (Table 1).

**Table 1:** Comparison of social demographic characteristics of 138 women with cancer of the cervix (cases) and 138 controls at Ocean Road Cancer Institute, Dar-es-Salaam, Tanzania

**Variable**	**Cases (138) ** **n (%)**	**Controls (138) ** **n (%)**	**p-value**
Age in mean years (SD)	51.9 (12.3)	47.4 (11.9)	0.002
			
Education*			
None	83(60.1)	55(39.9)	
Primary	50(36.2)	67(48.6)	<0.001
Secondary	3(2.2)	12(8.7)	
Higher learning	2(1.4)	4(2.9)	
			
Occupation			
Housewife	21(15.2)	28(20.3)	
Business	16(11.6)	24(17.4)	
Peasant farmer	92(66.7)	62(44.9)	0.06
Salaried employee	3(2.2)	13(9.4)	
Other	6(4.3)	11(8.0)	
			
Residence			
South	11(7.9)	7(5.1)	
Southern highland	20(14.4)	10(7.2)	
Eastern	49(35.5)	62(44.9)	0.13
Central	17(12.3)	10(7.2)	
Lake zone	18(13.0)	24(17.4)	
Northern	23(16.7)	25(18.1)	
			
Religion			
Catholic	36(26.1)	25(18.1)	
Protestant	30(21.7)	43(31.5)	
Born again	5(3.6)	6(4.3)	0.11
Muslim	48(34.8)	54(39.0)	
Other	19(13.8)	10(7.2)	
			
Marital status			
Single	2(1.4)	4(2.9)	
Married	94(68.1)	105(76.1)	0.62
Separated	12(8.7)	10(7.2)	
Widowed	30(21.7)	19(13.8)	

* Chi square for trend = 12.654

The mean parity of the cases (6.0, SD = 2.7) was higher than that of the controls (5.3, SD = 4.1) (Table 2). Women with cervical cancer were more likely to have ever smoked compared to the controls (OR = 5.3, 95% CI: 1.4–24.7). Other risk factors such as sexually transmitted infections (STIs), age at first coitus and use of oral contraceptives were not statistically associated with cancer of the cervix.

**Table 2:** Comparison of risk factors for cancer of the cervix among 138 cases and 138 controls at Ocean Road Cancer Institute, Dar-es-Salaam, Tanzania

**Variable**	**Cases (138) ** **n (%)**	**Controls (138) ** **n (%)**	**OR (95% CI)**
Age in mean years (SD)	51.9 (12.3)	47.4 (11.9)	1.0 (1.0 – 1.1)
Parity in mean births (SD)	6.0 (2.7)	5.3 (4.1)	1.1 (1.0 – 1.2)
Age at first sexual coitus in mean years (SD)	17.7 (4.0)	17.4 (2.1)	1.0 (0.9 – 1.1)
HIV positive			
No	109(79.0)	122(88.4)	1.0
Yes	29(21.0)	16(11.6)	2.0 (1.1 – 3.9)
Sexually Transmitted Infection			
No	108(79.3)	109(79.0)	1.0
Yes	30(21.7)	29(21.0)	1.0 (0.6 – 1.9)
Oral Contraceptives			
No	107(77.5)	111(80.4)	1.0
Yes	31(21.7)	27(19.6)	1.1 (0.6 – 2.0)
Ever smoked?			
No	128(92.8)	136(98.6)	1.0
Yes	10(7.2)	2(1.5)	5.3 (1.4 – 24.7)

Among the cases, 21.0% (29/138) were HIV-1 positive compared to 11.6% (16/138) of the controls. At bivariate analysis, HIV-1 positive status was associated with cervical cancer among the women (OR = 2.0, 95% CI: 1.1–3.9). At multivariate analysis, HIV-1 positive status was independently associated with cervical cancer (OR = 2.9, 95% CI: 1.4–5.9). Age and parity confounded the association between HIV-1 and cervical cancer, raising the odds ratio from 2.0 to 2.9. This effect was evident when age and parity were treated as either continuous or categorical variables. However, both age (OR = 1.02, 95% CI 1.00 – 1.05 per year) and parity (OR = 1.14, 95% CI 1.02–1.28 per birth) remained significantly associated with cervical cancer. Although education level was a confounder of the relationship between HIV-1 and cervical cancer when handled singly, raising the odds ratio from 2.0 to 2.3, it was not necessary to control for it once parity and age were controlled for. Smoking was significantly associated with cervical cancer (OR = 5.7, 95% CI: 1.1 – 28.9) but was not a confounder of the association between HIV-1 and cervical cancer (Table 3).

**Table 3:** Multivariate analysis of risk factors for cancer of the cervix among 138 cases and 138 controls at Ocean Road Cancer Institute, Dar-es-Salaam, Tanzania

**Variable**	**OR (95% CI)**
HIV positive	2.9 (1.4 – 5.9)
Age (per year)	1.02 (1.00 – 1.05)
Parity (per birth)	1.14 (1.02 – 1.28)
Ever smoked?	5.7 (1.1 – 28.9)

## Discussion

Our study showed that women with cervical cancer were three times more likely to be infected with HIV-1 than women without cervical cancer. Furthermore, the risk of cervical cancer increased with age, higher parity, and among those who had ever smoked. Our estimate for the association between HIV-1 and cervical cancer of OR = 2.9 is consistent with the risk or odds ratio range of 1.6 to 2.4 reported in previous studies. Few studies have established an association between HIV and invasive cancer of the cervix in sub-Saharan Africa [1,26,27,33,34]. To explain this situation, there are a number of plausible reasons.

First, though cancer of the cervix is the commonest malignancy in women in sub-Saharan Africa, it is rare when compared to many other diseases. Furthermore, invasive cancer of the cervix is less frequent than pre invasive cervical cancer. Second, it has been suggested that the combination of cancer of the cervix and HIV is usually lethal and many women with these two diseases die before they seek health care [35,36]. However, the evidence for this view is lacking, perhaps because of the difficulty of identifying such cases. In contrast, Wabinga and colleagues in an analysis of population based data linked to the cancer registry in Uganda showed that HIV did not influence survival of patients with invasive cervical cancer [37].

Third, methodological problems related to studies on invasive cervical cancer may have contributed to the situation. A review of the literature suggests that studies that have established an association between HIV and cancer of the cervix, in general, share certain characteristics. The common characteristics include the use of a population rather than a hospital setting [27,29]; use of the cohort or cross sectional study design as opposed to the case control design [26,27,29]; having large samples (usually thousands of participants) [26,27,29,33,38]; and the use of population controls [27,29]. It would therefore appear that methodological issues including the challenges of ensuring adequate power of studies (particularly since the association between HIV and cancer of the cervix is reported to be weak to modest), of minimizing bias, and of controlling for confounding may have contributed to the scarcity and negative findings of studies on invasive cervical cancer and HIV.

Confounding, in particular, is of special interest given the role of HPV in the aetiology of invasive cervical cancer. It has been established that HPV is the cause of invasive cervical cancer and it is believed that HPV negative cases of invasive cervical cancer should not exist if high quality methods for HPV detection are used [12,13,39]. Therefore, in a study to assess the association between HIV and invasive cervical cancer, it would be impossible to control for HPV because there would be *no HPV negative cases with invasive cervical cancer*. The implications of this are that HPV cannot be assessed as a confounder of the association between HIV and invasive cervical cancer. Instead, HIV should be assessed as a confounder for the relationship between HPV and invasive cervical cancer. However, even this approach appears to be futile because one of the prerequisites for such an assessment would be to establish an association between HIV and invasive cervical cancer *among the HPV negative cases of invasive cervical cancer who theoretically do not exist*.

Data on control for HPV among studies that have looked at HIV and invasive cervical cancer is very limited and also conflicting. Ter Meulen [31] and Moodley [28] controlled for HPV and found no association between HIV and cervical cancer. However, Hawes and colleagues found an association between HIV and cervical cancer after controlling for HPV [26]. Although all the studies were hospital based, the study by Hawes and colleagues was cross sectional (perhaps providing the same source population for cases and controls) and in addition had a much larger sample size.

In our study, we did not measure HPV and therefore we did not control for it in the analysis. It is possible that if we had measured it with currently available methods for detection of HPV and then adjusted for it, our estimate may have been weakened or even been nullified. This could perhaps be one of the reasons why Ter Meulen and Moodley were not able to demonstrate an association between HIV-1 and cervical cancer. Whether HPV should be adjusted for in the association between HIV-1 and cervical cancer or its precursors is an important epidemiological issue that requires further study. Our estimate was confounded by age and parity although the effect appears to be small. Although education was a confounder, it was not necessary to control for it because age and parity formed a minimum confounder group. Previous studies have reported that age and parity are independent risk factors for cervical cancer and are also associated with HIV-1 infection [9,10]. Consistent with previous studies, smoking was significantly associated with cervical cancer but was not a confounder because it was not associated with HIV-1 infection.

Although the mechanism by which HIV increases risk of cervical cancer is not completely understood, studies suggest that HIV-induced immunosuppression leads to an inability to control the expression of HPV and the production of HPV oncoproteins E6 and E7 [40,41]. According to Hawes and colleagues, this risk appears to be associated with increased HPV persistence that may result from immunosuppression related to HIV. Furthermore the risk is greater in women with CD4 counts less than 200 cells per microliter and in those with high plasma HIV RNA levels [19]. Studies have shown that HIV-1 infection is associated with an increased rate of HPV infection, mainly restricted to HR-HPV types which are the cause of invasive cancer of the cervix [16,18,26].

Women with higher parity were more likely to have cervical cancer than women of lower parity, with the risk of cervical cancer increasing by 1.1 times for each additional birth. The findings of our study confirm previous work that high-parity women are at high risk for cervical cancer [9,10]. It has been suggested that parity is a marker of the oestrogen-hormonal environment throughout the fertile years of women as well as a marker of repeated cervical trauma among highly parous women. It is not known whether hormones intervene in cervical carcinogenesis but oestradiol has been reported to induce immortalization of HPV infected cells [42].

Consistent with previous findings [11], our study showed that women who reported that they had ever smoked were six times more likely to have cervical cancer than women who had no history of smoking. Studies suggest that cancer causing chemicals (benzopyrene) from cigarette smoke damage the cervix and make it vulnerable to HPV infection. Women who smoke and also have HR-HPV genital infection are twice as likely to have pre-cancerous cells or to get cervical cancer.

Our findings have important implications for women with HIV or cervical cancer in Tanzania and other sub-Saharan countries which have high burdens of HIV and cervical cancer. According to Chirenje and colleagues, 95% of health care facilities in East, Central and Southern Africa have the infrastructure for cervical screening but the major bottlenecks to screening and treatment of cervical cancer are lack of policy guidelines, infrequent supply of basic materials, and lack of suitably qualified staff [7]. Ngwalle and colleagues have rightly pointed out the urgent need to develop a national policy and to introduce systematic screening for cervical cancer in Tanzania [43]. Such a task, important and urgent as it is, is no easy undertaking for a resource-constrained country like Tanzania.

However, the findings of our study suggest that a feasible alternative for Tanzania could be adoption of a high-risk approach that targets HIV positive women for cervical cancer screening. The resources for the cervical screening would initially come from the HIV/AIDS programmes. As has been pointed out, antiretroviral therapy (ART) for HIV provides a golden opportunity to improve cervical screening through the sharing of the ART resources and the frequent check-ups of women on ART that can also be used to screen for cervical cancer [44,45]. This approach would enable the Tanzania Ministry of Health and Social Welfare to develop the capacity for cervical screening and treatment in a gradual and feasible manner, while mobilizing additional resources and the population that would be essential for a national cervical cancer screening and treatment programme.

Our study had a number of potential limitations that may have distorted our estimates. Selection bias among the cases may have occurred in our study. The cases were selected from the only cancer treatment centre in Tanzania and thus were more likely to be representative, with regard to the exposure (HIV infection), of cervical cancers in the population than if they were selected from hospitals. However, a very small proportion of all cases of cervical cancer are seen in hospitals, and even fewer are referred to ORCI. The cases seen in ORCI are likely to be those who have access to health services (geographical, economic); women with advanced cancer; and possibly the survivors of HIV and cervical cancer.

In our study, the controls were mostly attendants or visitors of the cases. Attendants are usually female relatives who provide care for the patient while in hospital. The attendants or visitors usually came from the same community as the cases. Thus the controls in our study were "community" controls, derived from the same source population as the cases, and therefore representative of the non cervical cancer cases with regard to the exposure (HIV infection). To validate this, we found that the frequency of HIV-1 in the controls was similar to that in the general population. However selection bias may have occurred since the controls were significantly different from the cases in age, parity and education. Selection bias among the cases and controls could have caused an under-estimate of our findings.

Measurement bias may have occurred among the 20% of the controls who were not screened for cervical cancer leading to under-estimation. Similarly, women with pre-invasive disease could also have been included among the controls and caused under-estimation. Recall bias with possible differential misclassification and over or under-estimation of effect could have occurred in the assessment of age of first sexual coitus. Since there was no blinding, interviewer bias could also have occurred in the assessment of risk factors. However, we took precautions by training the interviewers and standardizing the instruments. We did not measure HPV and therefore did not control for it although as discussed earlier this could have been problematic.

## Conclusion

Notwithstanding these limitations, our study has demonstrated that HIV-1 infection is associated with invasive cancer of the cervix. Resource-constrained countries with a high burden of HIV and cervical cancer should adopt a high-risk approach that targets HIV-1 positive women for screening of cervical cancer initially by utilizing HIV/AIDS resources.

## Competing interests

The authors declare that they have no competing interests.

## Authors' contributions

CK participated in the conception, design, and implementation of the study, statistical analysis, interpretation and drafting of manuscript. JM and TN participated in study conception, design, implementation and interpretation of the study. HRW participated in study conception, design, and interpretation of the study. JNK participated in the conception, design, statistical analysis, and interpretation of the study. CASK participated in the conception, design, interpretation and drafting of manuscript. All authors read and approved the final manuscript.

## Pre-publication history

The pre-publication history for this paper can be accessed here:


